# Far-Field Subwavelength Straight-Line Projection/Imaging by Means of a Novel Double-Near-Zero Index-Based Two-Layer Metamaterial

**DOI:** 10.3390/ma14195484

**Published:** 2021-09-22

**Authors:** Reza Dehbashi, Taras Plakhotnik, Timo A. Nieminen

**Affiliations:** 1 Faculty of Engineering, Architecture, and Information Technology, School of Information, Technology, and Electrical Engineering, University of Queensland, St. Lucia, Brisbane, QLD 4067, Australia; 2 Faculty of Science, School of Mathematics and Physics, University of Queensland, St. Lucia, Brisbane, QLD 4067, Australia; taras@physics.uq.edu.au (T.P.); timo@physics.uq.edu.au (T.A.N.)

**Keywords:** double-near-zero metamaterials, electromagnetic scattering, electromagnetic theory, electromagnetic waves imaging, scattering

## Abstract

In this paper, for the first time, tuned near-zero-index materials are used in a structure for the long-distance projection of very closely spaced objects with subwavelength separation. Near-zero-index materials have never been used for subwavelength projection/imaging. The proposed novel structure is composed of a two-layer slab that can project two slits with a subwavelength separation distance to a long distance without diverged/converged interference of the two imaged waves. The two-layer slab consists of a thin double-near-zero (DNZ) slab with an obtained tuned index of 0.05 and thickness of 0.04λ_0_ coupled with a high-index dielectric slab with specific thicknesses. Through a parametric study, the non-zero index of the DNZ layer is tuned to create a clear image when it is coupled with the high-index dielectric layer. The minimum size for the aperture of the proposed two-layer slab is 2λ_0_ to provide a clear projection of the two slits. The space between the slits is λ_0_/8, which is five times beyond the diffraction limit. It is shown that, through the conventional methods (e.g., only with high-index dielectric slabs, uncoupled with a DNZ layer), it is impossible to clearly project slits at a large distance (~λ_0_) due to the diffraction limit. An analytical analysis, as well as numerical results in a finite-element-based simulator, confirm the function of the proposed structure.

## 1. Introduction

Metamaterials, whose permittivity or permeability is close to zero (generally called near-zero-index metamaterials), have recently been the focus of many research groups [1,2,3,4,5,6,7,8,9,10,11,12,13,14,15,16,17,18,19,20,21,22,23,24,25,26]. Metamaterials whose permittivity is close to zero are called epsilon-near-zero (ENZ) materials. If the permeability is close to zero, they are called mu-near-zero (MNZ) materials. Metamaterials with both permittivity and permeability close to zero are called double-near-zero (DNZ) materials or epsilon-and-mu-near-zero (EMNZ) materials [8,9]. In DNZ materials, the phase velocity of electromagnetic waves is much larger than the speed of light in vacuum, hence the wavelength of the wave is extremely large and the field is almost uniform. Such metamaterials have been used to manipulate the phase front of the electromagnetic waves [6] and to change the polarization of light [27]. They have also been used to achieve total transmission through subwavelength channels or bends [28]. Other applications include enhancing the radiation pattern of antennas [6,29], improving radiation efficiency of antennas [13,30], enhancing the scanning range of phase array antennas [31], improving matching for different structures [5], and manipulating transmission characteristics by embedding some defects in their structures [32]. They have also been employed for super-reflection or cloaking by embedding perfect electric conductors (PEC) or perfect magnetic conductors (PMC) in their structure [10,11]. Superscattering [33] and terahertz (THz) reflectarray antennas [34] are other examples of their applications.

Total transmission is another phenomenon that has been achieved in the last decade using near-zero materials. To achieve total transmission, some research used circular defects with precise shape, materials, and position for the defects inside the zero-index materials [32]. In [35], the near-zero materials were placed inside a rectangular waveguide. The waveguide had a particular shape in the middle where the near-zero material is located. The near-zero material was inserted between two materials of the same type inside the waveguide to achieve total transmission. In [11], perfect electric conductors (PECs) were used inside a DNZ material to allow the total transmission of the wave. In [10], a complex structure of inhomogeneous anisotropic media with a near-zero permittivity component was adopted for the tunneling of the wave.

This paper, using a total transmission technique, proposes a novel two-layer DNZ based slab that can project two slits separated by the distance of λ_0_/8 on a distance of about $\lambda_{0}$ ($\lambda_{0}$ is the wavelength in free space). This is the first time that zero-index materials are used in a structure to project very closely spaced objects with subwavelength separation. In addition, zero-index materials have not been used in the previously introduced lenses and they have employed other techniques. The reason is that the field inside zero-index materials is uniform, and normally a clear image of two closely spaced objects cannot be created through them. In this paper, the objects for imaging are two closely spaced slits in a conductor plane. Wave radiation through a single slit in a conductor plane causes diffraction, as has been shown in the literature [36]. Therefore, it has been a challenge to image two very closely spaced slits, particularly if they are separated by a distance of a fraction of a wavelength and if the images are at far-field distance. The reason is that the two images interfere with each other due to diffraction. The challenge of double-slit diffraction [36,37] has been addressed in our paper with the proposed novel two-layer structure that highly reduces the diffraction from two slits. The paper shows that one high-index dielectric slab alone cannot perform the imaging, and, therefore, it must be coupled with a tuned DNZ index layer.

It should be noted that the current paper is not introducing a lens, as lenses are for magnification and no magnification occurs in the introduced structure. The function of the proposed structure is the long-distance projection of very close objects in a direct line. The proposed two-layer slab consists of a high-index dielectric layer with a dielectric constant of 64 coupled with a thin, tuned DNZ-index layer. This paper shows that the dielectric layer with a constant of 64 without the presence of the DNZ layer cannot project the two slits without having the divergence of the two rays from the two slits. In practice, considering that the dielectric value of water is high in microwave frequencies (e.g., 81 @1GHz or 65 @10GHz, at room temperature) [38], for microwave frequency range applications, a slab with an effective dielectric value of 64 can be created by adding some holes to a water slab with the fraction of wavelength separation between the holes. The DNZ layer has a near-zero permittivity and permeability of 0.05 and thickness of 0.04λ_0_, which are obtained by a parametric study. If the index value is closer than 0.05 to zero, the wave inside the DNZ layer becomes uniform and therefore creates a blurred image. For the higher values of the index (higher than 0.05), the outgoing wave from the slits inside the DNZ layer is not directive enough to project the two slits clearly, as the waves from the two slits interfere with each other because of the diffraction. Additionally, the structure works better for a thin DNZ layer. When the DNZ layer is thick, there is enough space for rays coming from the two slits to diverge long enough to interfere with each other.

Without the proposed structure, if the space between two slits is less than about λ_0_/2 (0.61λ_0_ is the conventional diffraction limit in optical microscopy), the diffracted waves from the two slits will interfere with each other and create an unclear image if the intention is to project the slits to a distance of about λ_0_ or longer.

## 2. Process of the Design

The two-layer slab is designed to have total transmission when the DNZ layer is considered a perfect zero-index material and the incident wave is a plane wave without the presence of the two slits. This is called an ideal scenario. Therefore, total transmission is the condition of the initial design when the DNZ layer’s index is perfectly zero and the formulations are provided for that. This is explained in Section 3. In Section 4, the index of the DNZ layer is tuned with the presence of the slits, while the high-index dielectric layer’s design remains intact. It is shown that the designed structure works perfectly for the projection of the two slits. In the ideal scenario, the wave source is a plane wave; however, in the real scenario, with the presence of slits, each of the slits behaves in the manner of a line source, and a plane wave is not a wave source for the proposed two-layer slab. However, using the approximation of the ideal scenario, the designed structure works well for the distant projection of closely spaced slits with a distance that is below the diffraction limit, even though the plane wave is not the main source. The diffracted waves from the two slits are considered the main sources. The proposed structure can overcome the diffraction limit due to the very high numerical aperture (NA) of the two-layer slab, which is analytically and numerically shown in the next sections. Briefly, high NA is a result of the DNZ layer due to its high-wave directing nature, as well as the result of the high-dielectric constant of the second layer. It should be noted that single-zero materials such as ENZ cannot be used in this two-layer structure as they would be highly mismatched with free air. The proposed structure can be used in applications where the projection of subwavelength details at a distance is necessary.

## 3. Total Transmission Technique: Ideal Scenario

**Figure 1:**
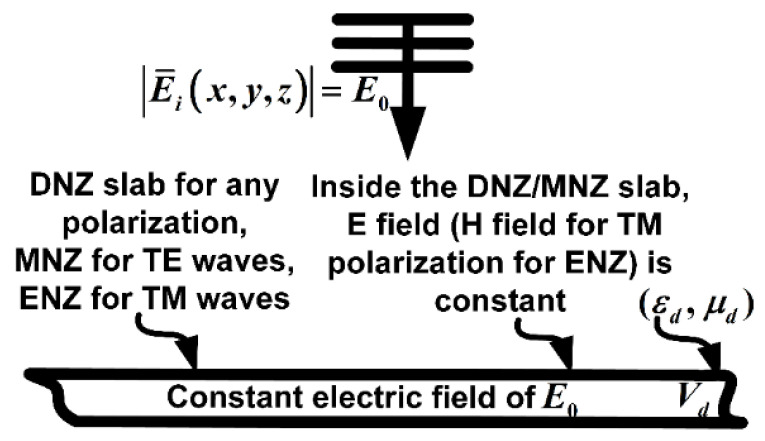
The entire DNZ slab has a constant electric field upon incident of fields with any polarization. For *TE* and *TM* polarized waves, the same happens if the material is MNZ and ENZ, respectively.

When a wave with any polarization passes through a DNZ material, the wave front of the outgoing wave adopts the shape of the outer surface of the DNZ material. In MNZ and ENZ materials, the wave front is tailored for the *TE* and *TM* polarized waves, respectively [6] (Figure 1).

Another interesting feature for DNZ materials is that the field inside them is uniform. For this to be analytically analyzed, it should be assumed that the electric field inside a near-zero slab with a volume *V_d_* is *Ē_d_* (Figure 1). Subsequently, the corresponding magnetic field is obtained by Equation (1):

(1) $${\bar{H}}_{d}=(1/i\omega \mu_{0}\mu_{d})\nabla \times {\bar{E}}_{d}$$

In the above equation *μ_d_* ≈ 0. Therefore, the electric field *Ē_d_* must be constant inside the entirety of the DNZ slab (volume *V_d_*) to have a finite value for *H_d_*.

Because the field inside the *V_d_* is constant, the Maxwell–Ampere law can be applied in that region when this type of material, coupled with a dielectric layer in the next part, is analyzed.

In this section, based on the above feature, the condition for total transmission for a DNZ slab coupled with a high-dielectric slab under a plane wave illumination is provided. Subsequently, in the next section, it is shown that the current design with slight adjustments is adequate for the projection of two slits with subwavelength distance. The adjustments include tuning the index value of the DNZ layer and its thickness while considering the minimum dielectric value for the dielectric slab. The minimum dielectric value among the values determined in this section is numerically obtained in Section 4.

**Figure 2:**
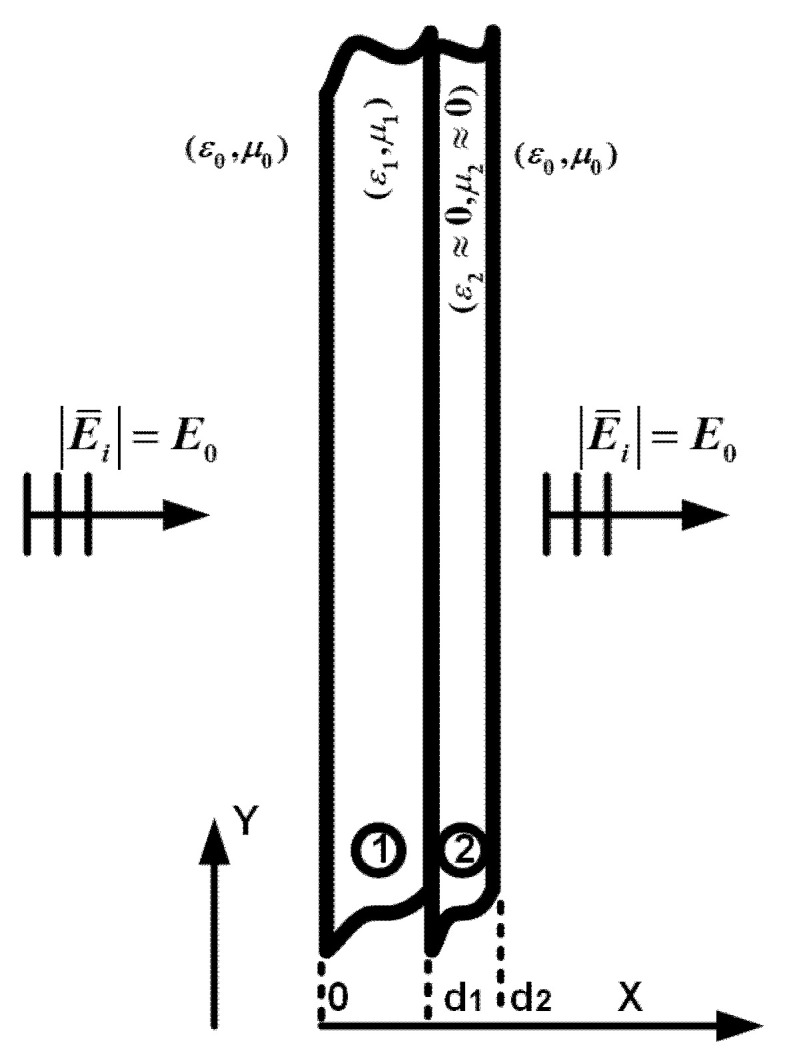
A DNZ slab coupled with a high dielectric slab. A plane wave with *TE_z_* polarization is incident on the structure perpendicularly.

Figure 2 shows a DNZ slab coupled with a dielectric slab. The wave behavior for this two-layer structure is analyzed, assuming a *TE_z_* polarized plane wave illuminates that:

For the *TE_z_* polarized wave on the left side, the fields are in the following form [39]:

(2) *x* < 0:
*E_z_^l^* = *E*_0_ (*e*^−*jk*^^0^*^x^* + *Re^jk^*^0^*^x^*)
*H_y_^l^* = *k*_0_*E*_0_ (*e*^−*jk*0^*^x^* − *Re^jk^*^0^*^x^*)/(*ωμ*_0_)

On the other side of the structure, the fields are:

(3) *d*_2_ < *x*:
*E_z_^r^* = *TE*_0_ *e*^−^*^jk^*^0^*^(x−d^*^2)^
*H_y_^r^* = *k*_0_*TE*_0_ *e^−jk^*^0^*^(x−d^*^2)^/(*ωμ*_0_)

where *k*_0_ is the wave number on the free space. After applying the boundary conditions and using the Maxwell–Ampere law, the transmission coefficient can be derived as:

(4) $$T\;=\;\frac{4}{\left(2-\frac{k_{0}\mu_{1}}{k_{1}\mu_{0}}-\frac{\mu_{0}k_{1}}{\mu_{1}k_{0}}\right)e^{-jk_{1}d_{1}}+\left(2+\frac{k_{0}\mu_{1}}{k_{1}\mu_{0}}+\frac{\mu_{0}k_{1}}{\mu_{1}k_{0}}\right)e^{jk_{1}d_{1}}}$$

where *k*_1_ is the wave number in the dielectric layer. To achieve total transmission, the thickness of the dielectric layer is obtained as:

(5) $$d_{1}=p\lambda_{1}/2$$

where *p* is an integer number and $\lambda_{1}$ is the wavelength of the wave inside the dielectric slab. It should be noted that the same result will be obtained if, in Figure 2, the field is normally incident from the right side. The thickness of the dielectric is 0.5λ_0_, with ε_r_ = 64, which is obtained from Equation (5) and is used in our design in the next section.

It should be noted that Equation (4) shows that the thickness of the DNZ slab has no effect on the transmission coefficient. This is also confirmed by our simulation results under plane wave illumination without the presence of the two slits. The phase velocity inside the DNZ slab is much faster than the phase velocity of light. Therefore, the wave passes across the slab very fast and, as a result, the thickness of the DNZ slab does not affect the total transmission. However, with the presence of the two slits, within the setup explained in the next section, it is concluded that a thin DNZ layer (~0.04λ_0_) provides more clarity for the projection of the two slits due to the diffraction caused by the two very closely spaced slits. For a thick DNZ layer, there is enough space for rays coming from two slits to diverge and interfere with each other. Therefore, a thin DNZ layer avoids this problem. It should be noted that the index used for DNZ in this paper is not zero, as we tune it in the next section; therefore, the field is not uniform inside the DNZ layer unlike the structure in Figure 1. By deviating the index from zero value, the field in the DNZ gradually starts to become non-uniform. Therefore, for a thick DNZ, the non-uniformity of the wave and diffraction cause unclarity of the projected waves coming from two slits.

## 4. The Proposed Two-Layer Structure

The principle of the design for the proposed two-layer structure without the presence of two closely spaced slits has already been explained. In this section, we explore and discuss the performance of the proposed structure, using analytical justifications and numerical results. In addition, after adding the two slits based on the numerical results, the index value of the DNZ layer is adjusted.

The two-layer slab is composed of two sections: a thin tuned DNZ-index layer and a high-index dielectric layer with specified dielectric values and thicknesses, as determined in the previous section. The DNZ layer operates as a lens. The other layer with high-index dielectric value is required as the complementary medium to the DNZ layer to increase its NA. The dielectric layer increases the NA by redirecting the refracted waves to the desired direction, which is close to the normal axis of the DNZ slab’s surface interface. For ideal DNZ slabs—i.e., a zero index slab—the outgoing wave is always normal to the interface, independent of the direction of incident wave; however, for our design, the refractive index of the DNZ layer is non-zero, as the requirement for the proposed two-layer. Due to non-zero values of permittivity and permeability of DNZ slabs (which in our design is tuned to be 0.05), the wave deviates from the normal axis at the interface. A high-index slab after the DNZ layer can correct this deviation and redirect the wave close to the normal axis.

The high resolution of the two-layer is justified as follows. The following formulation defines NA:

NA = *n* sin(*θ*) (6)

where *n* is the refraction index of the dielectric and *θ* is the angle of the incident field relative to the line perpendicular to the surface of the DNZ/dielectric interface. From (6), by increasing the dielectric index, the numerical aperture of the proposed two-layer structure is increased. NA, following Rayleigh’s resolution equation, has a direct relation with the resolution of the proposed two-layer:

Δy = 0.61λ/NA(7)

where Δy is the minimum distance that can be detected and λ is the wavelength of the illuminated field. Because the proposed two-layer slab has a very high NA, from Equation (7), it is concluded that its resolution is also very high. This is the reason that the proposed structure can function beyond the diffraction limit with very high resolution, as indicated below.

To examine the performance of the proposed two-layer structure for subwavelength projection, a setup is designed and simulated in a finite element (FEM)-based commercial EM simulator. In the setup, two slits in a PEC plate are placed close to each other. The PEC is placed at 0.06λ_0_ distance from the right side of the proposed two-layer slab. It should not be excessively close to the two-layer slab, as the numerical analysis shows that the level of the passed wave from the slits to the proposed structure will considerably drop. The space between the slits is λ_0_/8, which is nearly five times smaller than the diffraction limit of 0.61λ_0_. The lengths of the slits are arbitrary and chosen as nearly 0.2λ_0_. The projection/image line is positioned at 0.02λ_0_ distance from the left side of the high-index dielectric slab. The value of the dielectric layer and its thickness in the setup are 64 and 0.5λ_0_, respectively, which are obtained from Equations (4) and (5), respectively, as described in in Section 2. As discussed at the end of this section, the dielectric value of the dielectric layer should be more than 25 to achieve adequate resolution. The refractive index of the DNZ slab is tuned and obtained as 0.05. The reason for the tuning is that, if the index value of the DNZ layer is zero, the field inside the DNZ will be uniform (Figure 1), and, therefore, it cannot project the coming waves from two slits to the other side. If the index value is excessively high, the DNZ layer is not sufficiently directive to project the slits with clear resolution due to refraction. Therefore, the index of the DNZ layer must be tuned to find the best value to provide the highest resolution.

**Figure 3:**
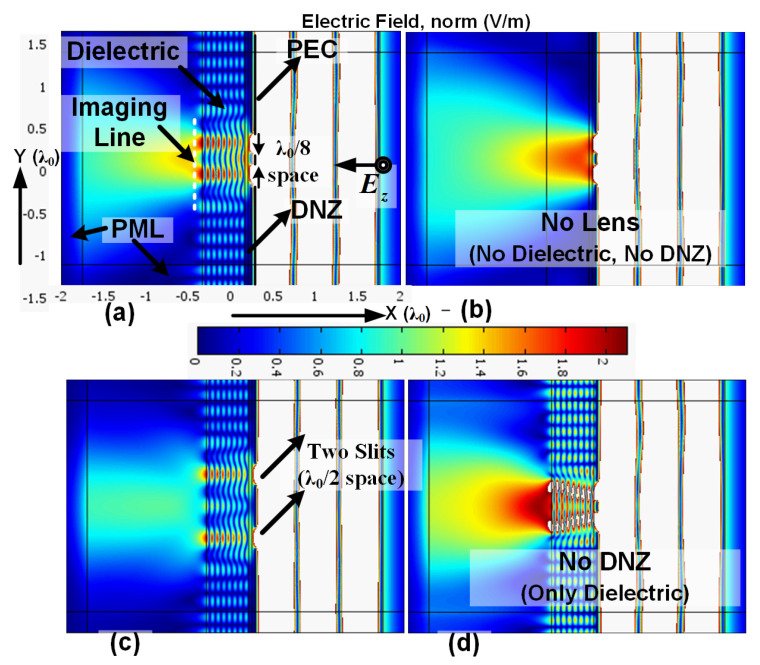
Projection of two PEC slits: (**a**) with the proposed two-layer slab—space between two slits: λ_0_/8; (**b**) without the two-layer slab; (**c**) with the two-layer slab—space between two slits: λ_0_/2; (**d**) only with the dielectric layer and without the DNZ layer—space between two slits: λ_0_/8.

The simulation for the setup, using the proposed two-layer slab, is shown in Figure 3a.

**Figure 4:**
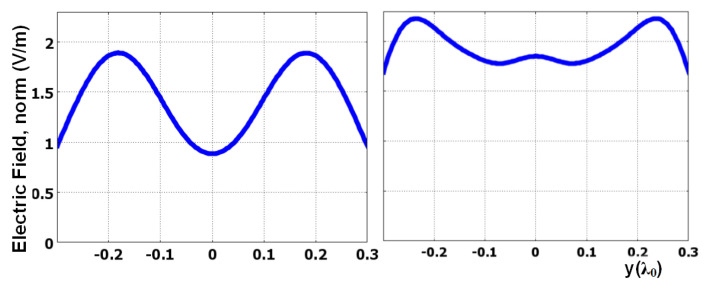
Electric field at 0.02$\lambda_{0}$ from the dielectric surface for cases (a) and (d) in Figure 3 (left and right panels, respectively).

The image line is a white dotted line shown on the left side of the dielectric layer in Figure 3a. As shown, the projection of the two slits on the image line is clear and distinctive. Figure 3b shows the field distribution without the presence of the two-layer slab. Without the two-layer slab, there is no clear image of the slits on the image line due to the diffraction limit. Figure 3c shows the clear image of the slits when the distance between slits is larger and close to the diffraction limit, i.e., 0.5λ_0_. Figure 3d shows the image without the DNZ slab. It shows that the high-index dielectric slab without the DNZ slab cannot hold the wave in a direct way and the wave slightly diverges. This is probably because the wave is highly diffracted from the two slits and there is no DNZ slab to redirect them. Therefore, the presence of the DNZ slab is crucial for a long projection of this nature. Additionally, as shown in Figure 4, the setup in Figure 3a is highly advantageous in comparison with that in Figure 3d, even if the divergence of the wave in Figure 3d is ignored. Figure 4 shows that, for case (a), the levels on the sides are 1.9 V/m, while that in the middle is 0.9 V/m (more than double contrast). For case (d), the levels on the sides are 2.2 V/m, while that in the middle is 1.8 V/m, which shows less contrast compared to case (a).

The role of the DNZ and dielectric layers can also be explained by Snell’s law of diffraction by approximation, in which one diffracted ray from the slits is chosen. For that ray, Snell’s law is n_1_.sin(*θ*_1_) = n_2_.sin(*θ*_2_), where n_1_ and n_2_ are the refractive indices of DNZ and of the high-index dielectric slabs, respectively. The angles *θ*_1_ and *θ*_2_ are the angles of the ray in the DNZ and the dielectric slabs with normal axis, respectively. If n_1_ was zero, the angle *θ*_2_ would have been zero, too, which means that, for a perfect zero-index slab, the wave on the right side would be normal in relation to the interface, regardless of the material of the right side (n_2_). However, for a DNZ slab, n_1_ is not exactly equal to zero. Therefore, if *θ*_1_ is high, due to an oblique incidence, to achieve close-to-zero value for *θ*_2_, n_2_ should be high. For instance, if n_1_ = 0.1 and sin(*θ*_1_) = 0.9 (*θ*_1_ = 65 degrees), and the other side is free space (n_2_ = 1), then, from Snell’s law, the outgoing wave is inclined with angle *θ*_2_ = 5°. By replacing the free air with a high-index material with n_2_=8 (er = 64) on the right side of the DNZ lens, *θ*_2_ equals 0.6°, which is almost normal to the interface (highly directive wave).

**Figure 5:**
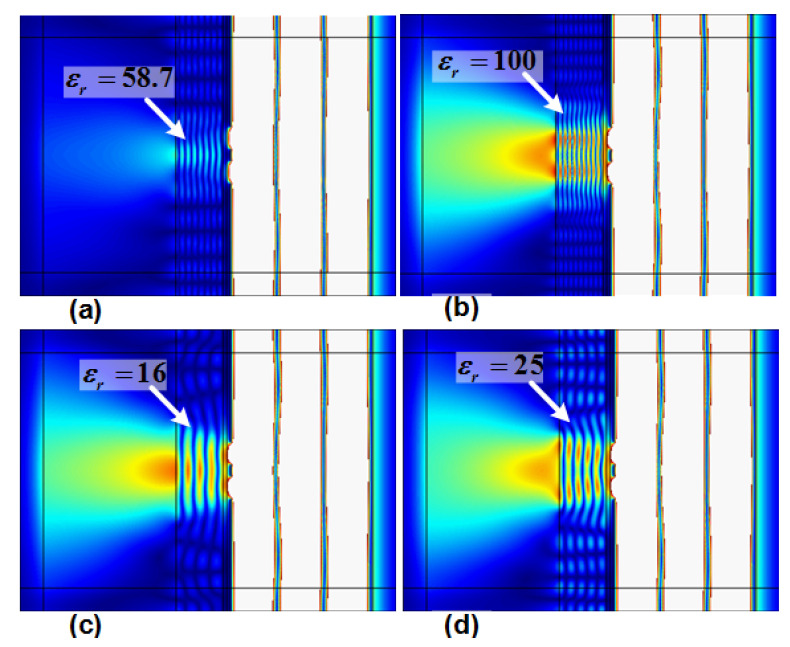
Projection of the two slits with the proposed two-layer structure for various values for the dielectric layer: (**a**) ε_r_ = 58.7; (**b**) ε_r_ = 100; (**c**) ε_r_ = 16; (**d**) ε_r_ = 25.

Figure 5 shows the projection of two slits for different values for the dielectric slab. From Equation (4), the transmission of the incident wave from the two-layer structure for some values of dielectric is maximum (total transmission), i.e., one, while for some values, it is minimum (~0.2). From Equation (4), the values of the dielectric constant of 100, 25, and 16 for the dielectric slab of width 0.5λ_0_ under plane incident wave cause total transmission (obtained from ideal scenario). For these values of the dielectric constant, as shown in Figure 5b–d, the maximum of the field passes through the slits and two-layer structure. From Equation (4), it can be determined that the dielectric value of 58.7 blocks most of the wave, which is also evident in Figure 5a. This confirms that the approximation method explained in Section 2 is effective for the current design, as the dielectric values obtained for the ideal scenario of Figure 2 can function well for the current setup in Figure 3 after considering the other previously discussed requirements.

In addition to testing the validity of Equation (4) for the real scenario, Figure 5 is used to determine the minimum value for the dielectric constant in the two-layer design among the obtained values of 100, 64, 25, and 16; Figure 3a shows the result for the dielectric value of 16. Comparing Figure 3a and Figure 5 shows that the minimum value for the dielectric layer is 64, since, if the value falls below 64, there is no distinctive resolution of the two slits on the image line and the two rays diverge.

**Figure 6:**
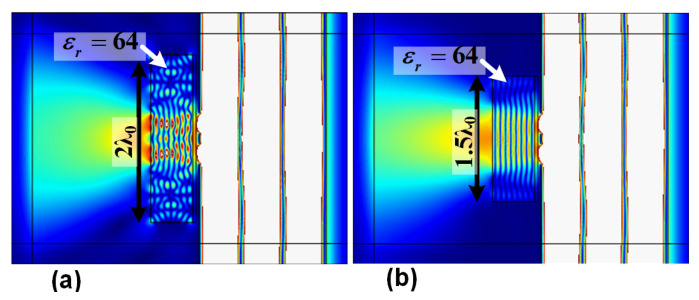
Projection of the two slits when the length of the proposed two-layer structure is (**a**) 2λ_0_ and (**b**) 1.5λ_0_.

Another parameter study is carried out to find the minimum required length for the proposed two-layer slab for resolution up to five times beyond the diffraction limit. For the current design, the dielectric value of the dielectric layer is assumed to be 64 with the thickness of 0.5λ_0_ (the design presented in Figure 3a). For these parameters, the parametric study shows that the minimum size of the aperture for the two-layer structure is 2λ_0_. Figure 6b shows the function of the two-layer slab for the length 1.5λ_0_. As shown, for length of 1.5λ_0_, the image of two slits is not clear and distinctive.

Finally, the numerical analysis shows that a dielectric layer that is excessively thick (~1.5λ_0_) will make the wave inside the dielectric layer too perturbed to obtain a clear image on the other side. For a thin dielectric layer, if the dielectric constant is ultra-high with the thickness provided by Equation (4), the projection of the two slits is clear even without the DNZ layer; however, since the dielectric is thin, the projection in not possible at a large distance (~λ_0_). It should be noted that the image line is almost on the dielectric layer, and, therefore, the projection distance depends on the thickness of the proposed two-layer slab, including the dielectric layer. When the DNZ layer is coupled with a thick dielectric layer, it is possible to create a clear projection at a longer distance compared to a thin dielectric layer, which is not possible with only dielectric layer without the DNZ layer. A two-layer structure, therefore, is proposed for the subwavelength projection. The above-mentioned restriction on the long-distance projection is due to the diffraction (that is, the diverged/diffracted wave from two slits) that, for a thick material, causes the perturbation and interference.

## 5. Conclusions

In this paper, a two-layer structure with 2λ_0_ length and high numerical aperture (NA) is proposed to project two slits with subwavelength separation at a long distance. The minimum distance between the slits is nearly five times beyond the diffraction limit. The slab consists of a thin DNZ layer with an index of 0.05 coupled with a high-index dielectric slab with a dielectric value of 64. Analytical and numerical analyses for the two-layer design to project two closely spaced slits support each other.

## Data Availability

Not applicable.
